# A systematic review and meta-analysis of the association between maternal polycystic ovary syndrome and neuropsychiatric disorders in children

**DOI:** 10.1038/s41398-021-01699-8

**Published:** 2021-11-08

**Authors:** Pallavi Dubey, Bhaskar Thakur, Sheryl Rodriguez, Jessika Cox, Sheralyn Sanchez, Anacani Fonseca, Sireesha Reddy, Deborah Clegg, Alok Kumar Dwivedi

**Affiliations:** 1 Department of Obstetrics and Gynecology, Paul L. Foster School of Medicine, Texas Tech University Health Sciences Center El Paso, El Paso, TX USA 79905; 2 Division of Biostatistics and Epidemiology, Department of Molecular and Translational Medicine, Paul L. Foster School of Medicine, Texas Tech University Health Sciences Center El Paso, El Paso, TX USA 79905; 3 Graduate School of Biomedical Sciences, Texas Tech University Health Sciences Center El Paso, El Paso, TX USA 79905; 4 Department of Pediatrics, Paul L. Foster School of Medicine, Texas Tech University Health Sciences Center El Paso, El Paso, TX USA 79905; 5 Office of Research, Texas Tech University Health Sciences Center El Paso, El Paso, TX USA 79905; 6 Department of Internal Medicine, Paul L. Foster School of Medicine, Texas Tech University Health Sciences Center El Paso, El Paso, TX USA 79905

**Keywords:** Autism spectrum disorders, Predictive markers, Scientific community

## Abstract

There is emerging evidence demonstrating an association between maternal polycystic ovary syndrome (PCOS) and autism spectrum disorder (ASD) in children, however, the cumulative effect of maternal PCOS on the development of ASD or other neuropsychiatry disorders (NPD) in children and separately for males and females has not been examined. We sought to systematically evaluate the influence of maternal PCOS on a wide range of NPD including ASD, attention deficit hyperactivity disorder (ADHD), chronic tic disorder (CDT), other behavior disorders, anxiety, depression, bipolar disorder, schizophrenia in children as well as in women of reproductive age only. We queried electronic databases including PubMed, EMBASE, and Google Scholar, until March 2021. We used DerSimonian and Laird (D-L) random effects method to compute pooled effect size in terms of odds ratio (OR). Nineteen studies (1667851 mothers, 2260622 children) were included in this study. Mothers with PCOS had an increased odds of children diagnosed with ASD (OR = 1.40, *p* < 0.001), ADHD (OR = 1.42, *p* < 0.001), CTD (OR = 1.44, *p* = 0.001), anxiety (OR = 1.33, *p* < 0.001), as well as other behavioral symptoms (OR = 1.45, *p* < 0.001) in the adjusted analysis. The association between maternal PCOS and ASD (OR: 1.43 vs. 1.66), ADHD (OR: 1.39 vs. 1.54), and CTD (OR: 1.42 vs. 1.51) was found to be significantly consistent between males and females, respectively. Our data do not suggest increased fetal testosterone exposure is associated with increased autistic traits in children. However, PCOS was significantly associated with increased odds of a wide range of NPD in women themselves. Maternal PCOS is a risk factor for various NPD with a similar extent in their children regardless of their underlying comorbidities. Managing PCOS is essential for women’s health as well as for their children’s health. More research is needed to determine the mechanisms and links between maternal PCOS and NPD in children.

## Introduction

Early diagnosis and management of neuropsychiatric disorders (NPD) such as autism spectrum disorder (ASD), attention deficit hyperactivity disorder (ADHD), chronic tic disorder (CTD), anxiety, and other behavioral disorders in the pediatric population are clinically challenging. These disorders are often undiagnosed or misdiagnosed in children and youth due to variations of signs and symptoms. The prevalence of these mental disorders ranges from 0.1% to 24% in children and youth [1, 2]. Currently, diagnosis of pediatric NPD has been characterized genetically, in addition, however, data suggest unhealthy behaviors including lack of physical activity, excessive or binge drinking, smoking, fair or poor nutritional status, and psychological health during a pregnancy have also been associated with NPD in their children [3–5]. However, given the costs and determents of these diagnoses, it is critical to identify risk factors and mechanisms which cause NPD in children in order to provide early prevention, screening, therapeutic advancement, and overall management. Emerging evidence suggests maternal polycystic ovary syndrome (PCOS) could be a potential risk factor for the development of various NPD in children. A small level meta-analysis [6] based on only seven studies showed a strong association between maternal PCOS and ASD in offspring. However, this study did not assess the effect of maternal PCOS on the development of ASD in children using the analysis of cohort studies. Moreover, the combined effect of maternal PCOS on the occurrence of various NPD other than ASD in children by combining data from individual studies has not been assessed in previous studies.

PCOS occurs in 1.5–26% of all reproductive-aged women among different ethnic/racial populations [7]. This endocrine disorder is associated with type II diabetes, cardiovascular disease, and endometrial carcinoma. Most PCOS women have obesity and metabolic disorders along with infertility, pregnancy complications, and overall poor quality of life. The probable underlying mechanism for the linkage between PCOS and ASD is the sex steroid testosterone, a major contributor for PCOS females. High levels of prenatal testosterone exposure are also associated with autistic conditions in children and may affect permanent structural, epigenetic, or molecular changes. Maternal pathophysiological conditions including circulating inflammatory markers and familial environmental and nutrient factors may also influence metabolic conditions and NPD in children [8, 9]. Emerging evidence from neuroimaging studies reported elevated fetal testosterone levels impact grey matter volume in sexually dimorphic brain regions associated with structural and functional changes in brain development of autism and other psychopathologic conditions [10, 11]. Even minimal hormonal imbalances due to activation of hormone receptors in mothers may exert a detrimental effect on the growth of the fetal brain contributing to neurodevelopmental conditions [6, 12].

Although some studies have shown an association between maternal PCOS with ASD in children, other studies have yielded no association [13–15]. Recently, several studies have examined the relationship of maternal PCOS with other NPD including ADHD, CTD, anxiety, and other behavior/mental disorders in children regardless of sex [8, 9, 16–19]. Some individual studies [8, 9, 16–18, 20, 21] reported children with multiple disorders allowing us to assess the influence of maternal PCOS on multiple disorders in children. Moreover, the pooled association of maternal PCOS with any specific NPD in children was not assessed according to sex in any studies. In addition, several studies reported contradictory correlations between elevated perinatal testosterone exposures with autistic traits [11, 22–26]. Using a systematic review and meta-analysis, our study investigated the combined evidence for the association of maternal PCOS and ASD, ADHD, CTD, other behavior problems, anxiety, depression, bipolar disorder, and schizophrenia in children. Since most of the studies [8, 9, 15, 16, 18, 19, 21, 27, 28] included in this meta-analysis adjusted unhealthy behaviors and other critical risk factors confounding the association between maternal PCOS and NPD in children, we estimated pooled associations based on unadjusted and adjusted estimates separately. We also evaluated the influence of maternal PCOS on the development of ASD, ADHD, and CTD in males and females separately and examined the association between fetal hyperandrogenic exposures with autistic trait scores. In addition, we quantitatively synthesized the cumulative relationship of PCOS with a range of NPD in women of reproductive age only. The cumulative association was assessed by updating the recent evidence for the association between PCOS and NPD within women themselves.

## Materials and methods

We followed the Preferred Reporting Items for Systematic Reviews and Meta-analyses (PRISMA)-2020 guidelines, the Meta-analysis of Observational Studies in Epidemiology (MOOSE), and SAMBR guidelines in conducting and reporting this study [29–31].

### Data source and search strategy

A comprehensive search on PubMed and EMBASE was made for any studies reporting data on maternal PCOS and neuropsychiatric symptoms in children prior to March 30, 2021. In addition, a google scholar search was also made up to five pages to determine any relevant study for this meta-analysis. The following search terms: (“maternal polycystic ovary syndrome” OR “polycystic ovarian syndrome” OR “PCOS” OR “fetal testosterone”) AND (“neuropsychiatric disorder” OR “neurodevelopmental disorder” OR “mental disease” OR “autism” OR “ASD” OR “attention deficit disorder” OR “ADHD” OR “eating disorder” OR “depression” OR “anxiety” OR “schizophrenia” OR “bipolar disorder”) AND (“offspring” OR “children” OR “child”) were used to screen the eligible articles. Full articles and abstracts were independently reviewed for the eligibility criteria by three authors (PD, SR, and JC). References from the review articles were also cross-checked to screen any pertinent studies for this study. We included any articles evaluating the association between maternal PCOS with NPD in children regardless of the direction of the study. Study selection was also restricted to articles written in English only. Descriptive studies (case series, case reports), clinical trials, non-human studies, abstract, review, and duplicated studies were excluded from this study.

### Inclusion and exclusion criteria

Any studies included children that (a) were diagnosed with ASD or other NPD using International Classification of Diseases (ICD) codes or Diagnostic and Statistical Manual of Mental Disorders (DSM-IV), read code, or any other validated instruments for diagnosing different mental disorders, (b) reported prenatal hormone or fetal testosterone exposures using fetal amniotic measurements or umbilical cord blood samples or maternal plasma levels (c) had mothers with self-reported PCOS or PCOS diagnosed with Rotterdam, National Institute of Health (NIH) criteria or any other diagnostic methods including ICD codes, read codes or other established criteria were included. Only studies reporting sufficient data to compute an association index (odds ratio or correlation coefficients) were included in the analyses.

### Data extraction and synthesis

Three authors (PD, JC, and SR) independently reviewed each article and extracted relevant data pertaining to this study. To reduce the risk of duplication or bias, an investigator (BT) double-checked the compiled data and proofread them for accuracy. Disagreements were resolved by discussion with senior authors (SR, DC, and AD). We prepared a comprehensive datasheet to extract relevant data for this study. Specifically, we extracted the following key information from the eligible studies: (a) study characteristics (title, name of the first author, country, year of publication, study design, sample size (child), sample size (women), gender ratio, mean age of the child, maternal age means, comorbidities (gestational diabetes, pre-eclampsia, and others) and follow up years if any) (b) neuropsychiatric outcome characteristics (ASD or autistic trait scores, ADHD, CDT, anxiety, schizophrenia, bipolar disorder, depression, and other behavior problems) of the children and mothers, and (c) exposure characteristics including PCOS diagnosis or testosterone levels. The primary outcome considered in this study was the individual NPD in children. The other secondary outcomes were the presence of individual NPD in women themselves and autistic trait scores in children.

### Quality assessment

Four independent reviewers (PD, JC, SR, and BT) assessed the quality of the studies using the Newcastle–Ottawa Scale applicable for the evaluation of non-randomized studies. Accordingly, each study receives a score that is based on the composition of scores from three domains of the methodology (a) subject selection (b) comparability of cases and controls (c) ascertainment of exposures in case-control studies and outcomes in cohort studies. The overall score ranges between 0 and 9 stars. The overall score is converted into good, fair, or poor quality score according to the Agency for Healthcare Research and Quality (AHRQ) standards. Any disparities in scores were further resolved by a senior author (AD).

### Statistical analysis

For the quantitative assessment, odds ratios (ORs) and their 95% confidence intervals (CIs) were calculated as the effect size estimates to measure the association between maternal PCOS with NPD in children and in women with PCOS themselves. Most of the studies included in the meta-analysis reported the adjusted associations after adjusting the well-known risk factors of mothers and children for various outcomes. However, the adjusted factors varied across the studies and thus we estimated pooled associations based on unadjusted and adjusted ORs separately. For studies where the ORs were not reported, they were calculated from available frequencies. The correlation coefficient (r) was calculated to assess the strength of the linear relationship between fetal testosterone exposure and autistic traits in children. If the correlation coefficients were not reported, appropriate conversion was made from the standardized mean difference [32]. An I^2^ statistic was used to assess heterogeneity in estimates between studies and *I*^*2*^ > 50% indicates a significant presence of heterogeneity [33]. The DerSimonian and Laird random-effects model was used to obtain a pooled estimate of association. The association of PCOS mothers with ASD or ADHD in children was further summarized separately according to study characteristics such as study design (case–control and cohort), study sample (population-based or hospital-based), and geographic location (USA, UK, others). Sensitivity analysis was carried out to assess the influence of study characteristics and subject characteristics on the pooled associations. Specifically, we restricted studies in sensitivity analyses by exposure definition (PCOS criteria including hyperandrogenemia, self-reported PCOS), out**c**ome definition (ASD criteria), quality index, and effect size estimation. We also explored to identify any maternal characteristics associated with ASD or ADHD estimates using meta-regression analyses. To assess the publication bias, funnel plot, Begg’s and Egger’s tests were used. We presented results with forest plots and summarized with OR, 95%CI, and two-sided *p* values. *P* values less than 5% were considered statistically significant results. All analyses were carried out using STATA V.15.1 (StataCorp. 2017. Stata Statistical Software: Release 15. College Station, TX: StataCorp LLC.). The STATA codes for statistical analyses and the dataset for the current study are available from the corresponding author on reasonable request.

## Results

### Search results

**Fig. 1:**
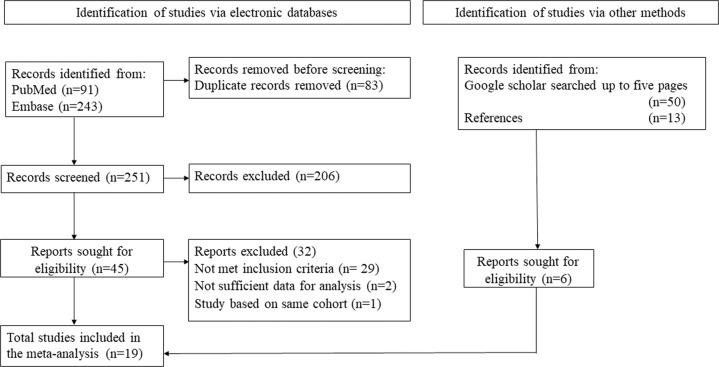
The selection process of studies for meta-analysis. PRISMA flowchart. PRISMA Preferred reporting items for systematic reviews and meta-analyses.

Our search yielded 340 articles from various databases including PubMed, EMBASE, and additional sources. After a critical review of all articles, only 51 articles met the initial eligibility criteria and were fully assessed for their eligibility in this systematic review. Among 51 articles, 20 articles met the eligibility criteria. However, one article [34] was excluded as this study was a sub-cohort of another included study [11] in this meta-analysis. Details of the search results are presented in the PRISMA flowchart (Fig. 1). Finally, 19 studies with 1,667,851 mothers and 2,260,622 children were included in our quantitative synthesis and evaluated for their quality assessment.

### Study characteristics and qualitative synthesis

Of 19 studies, 14 studies (*n* = 1,393,858) which included eight studies with a case–control design and six studies with a cohort design were used to determine an association between maternal PCOS (PCOS: 63,755 and without PCOS: 1,330,103) and individual NPD in children. The other five studies (three cross-sectional studies, one case–control study, and one cohort study) were included to evaluate the association between prenatal exposure to testosterone with autistic traits in children (*n* = 1,168). In addition, three studies also yielded data for determining an association between PCOS and NPD in women of reproductive age. The data from these three studies were also combined with other studies through additional search reporting the association between women with PCOS and NPD in women themselves. Most of the studies were population-based (11 studies) and from UK (5) or other European countries (four from Sweden, one from Italy, one from Denmark, and one from Finland), followed by the USA (four studies), Asia (one from India and one from China), and one from Australia. The studies used well-established criteria for PCOS diagnosis, one study used hyperandrogenism, three studies included self-reported PCOS, and four studies did not report any diagnostic criteria (Table 1). The range of average age of children and mothers was 1.6–22 years and 20–43.4 years respectively. The average length of follow-up for developing NPD was 0–27 years (Table 1). The reported range of obesity (14.3–49.3%), any psychiatric diseases (2–41%), and diabetes (3.5–3.85%) was observed as the most common comorbidities in maternal PCOS. The most commonly reported problem was infertility (24%), gestational diabetes (range: 2.6–22.5%), and pre-eclampsia (range: 0.01–4.2%) among PCOS mothers.

**Table 1:** Study characteristics.

Author (year)	Association	Country	Study design	PCOS criteria	Neuropsychiatric conditions	Total sample size (children)	Total sample size (mothers)	Total sample size (women)	Male/Female (child)	Mean age of child in years (SD)/range
Ingudomnukul et al. (2007) [13]	Maternal PCOS and NPD children (PCOS prediction) and within women (PCOS prediction)	UK	Case–control	TMQ	ASD	257	257	237	NA	NA
Auyeung et al. (2009) [22]	Fetal testosterone vs autistic traits in children	UK	Cross-sectional		ASD	235	235	NA	118/117	8.91(0.95)
Auyeung et al. (2010) [23]	Fetal testosterone vs autistic traits in children	UK	Cross-sectional		ASD	129	129	NA	66/63	1.60 (0.13)
Whitehouse et al. (2012) [25]	Prenatal testosterone exposure in umbilical cord and NPD children (NPD prediction)	Australia	Cross-sectional		ASD	374	366	NA	190/184	19–20 (range)
Palomba et al.(2012) [14]	Maternal PCOS and NPD children (NPD prediction)	Italy	Case–control	HA	ASD	75	75	NA	38/37	NA
Mamidala et al. (2013) [12]	Maternal PCOS and NPD children (PCOS prediction)	India	Case–control	Self-reported	ASD	942	942	NA	754/188	2–10 (range)
Xu et al. (2013) [26]	Fetal testosterone vs autistic traits in children	China	Case–control		ASD	109	109	NA	NA	control: 4.69 (1.19); case: 4.51 (1.71)
Baron-Cohen et al. (2015) [11]	Fetal testosterone vs autistic traits in children	Denmark	Cross-sectional		ASD	345	345	NA	Only male	NA
Kosidou et al. (2016) [19]	Maternal PCOS and NPD children (PCOS prediction)	Sweden	Case–control	ICD	ASD	232544	232544	NA	162639/69905	NA
Kosidou et al. (2017) [28]	Maternal PCOS and NPD children (PCOS prediction)	Sweden	Case–control	ICD	ADHD	558910	558910	NA	383319/175591	NA
Lee et al. (2017) [35]	Maternal PCOS and NPD children (NPD prediction)	Sweden	Case–control	ICD	ASD	739	739	NA	NA	NA
Schieve et al. (2017) [15]	Maternal PCOS and NPD children (PCOS prediction)	USA	Case–control	Self-reported	ASD	1538	1538	NA	1008/530	2–5 (range)
Berni et al. (2018) [17]	Maternal PCOS and NPD children (NPD prediction)	UK	Cohort	Read Code	ASD; ADHD	17847	17847	33876	NA	NA
	Within women	UK	Cohort	Read Code	ASD;ADHD; Schizophrenia; Anxiety; Depression	17668	17668	32710	NA	NA
Cherskov et al. (2018) [21]	Within women(PCOS prediction)	UK	Case–control	Read Code; NIH; Rotterdam	ASD	NA	NA	5826	NA	NA
	Within women(NPD prediction)	UK	Case–control	Read Code; NIH; Rotterdam	ASD; Schizophrenia; Anxiety; Depression	NA	NA	156980	NA	NA
	Maternal PCOS and NPD children(NPD prediction)	UK	Case–control	Read Code; NIH; Rotterdam	ASD	49715	49715	49715	25872/23843	10 (estimated from mothers age)
Bell et al. (2018) [16]	Maternal PCOS and NPD children (NPD prediction)	USA	Cohort	Self-reported	ASD; Other behavioral problem	5388	4453	NA	2302/2151;No informationfor 935 children	2.2 (1.09)
Hisel-Gorrman et al. (2018) [27]	Maternal PCOS and NPD children (PCOS prediction)	USA	Cohort	ICD	ASD	35040	35040	NA	27997/7043	2–18 (range)
Cesta et al. (2020) [8]	Maternal PCOS and NPD children (NPD prediction)	Sweden	Cohort	ICD	ASD; CTD;ADHD	239099	154376	NA	122247/116852	9.2 (7.6)
Chen et al. (2020) [18]	Maternal PCOS and NPD children (NPD prediction)	Finland	Cohort	ICD	ASD; CTD;ADHD; Other behavioral problem	1097753	590939	NA	561266/536487	4–22 (range)
Robinson et al. (2020) [9]	Maternal PCOS and NPD children (NPD prediction)	USA	Cohort	Self-reported	ADHD, Anxiety, Behavioral problem	1915	1624	NA	1017/898	(7–8)range
Total						2,260,622	1,667,851	279,344	1,288,833/933,889	1–22 years

Author (year)	Women with PCOS	Mothers with PCOS	Children with ASD	Mothers with ASD	Women with ASD	Children with ADHD	Women with ADHD	Children follow-up in years (range/median)	Maternal age mean (SD)/IQR/% of women in age range	Eligible for quantitative synthesis
Ingudomnukul et al. (2007) [13]	6	10	NA	54	1	NA	NA	NA	No ASD: 43.4 (6.1);ASD: 40.3 (6.3)	Yes
Auyeung et al. (2009) [22]	NA	NA	NA	NA	NA	NA	NA	6–11	Combined group:35.77 (4.40)	Yes
Auyeung et al. (2010) [23]	NA	NA	NA	NA	NA	NA	NA	1.5–2	Combined group:35.67 (4.21)	Yes
Whitehouse et al. (2012) [25]	NA	NA	NA	NA	NA	NA	NA	17	Combined group: 20–34 years: 78%	Yes
Palomba et al.(2012) [14]	NA	30	NA	NA	NA	NA	NA	NA	No PCOS: 36.4 (4.6); PCOS: 38.5 (4.2)	Yes
Mamidala et al. (2013) [12]	NA	13	58	NA	NA	NA	NA	NA	Combined group: 27	Yes
Xu et al. (2013) [26]	NA	NA	62	NA	NA	NA	NA	NA	control: 30.54 (3.03); case: 31.02 (4.83)	Yes
Baron-Cohen et al. (2015) [11]	NA	NA	NA	NA	NA	NA	NA	11–17	Control: 33.59 (5.03); ASD: 34.12 (5.89); Asperger’s: 33.52 (5.34); PDD-NOS: 32.32 (6.15)	Yes
Kosidou et al. (2016) [19]	NA	1,006	23,748	NA	NA	NA	NA	0–27	No ASD: 28.74 (5.10); ASD: 28.72 (5.54)	Yes
Kosidou et al. (2017) [28]	NA	2,341	13,540	NA	NA	NA	NA	0–27	No ADHD: 28.77 (5.06); ADHD: 27.65 (5.43)	Yes
Lee et al. (2017) [35]	NA	120	122	NA	NA	NA	NA	0–27	NA	Yes
Schieve et al. (2017) [15]	NA	197	629	NA	NA	NA	NA	NA	20–34 (70.3%)	Yes
Berni et al. (2018) [17]	7,976	8,962	165	30	NA	137	24	control: 2.81 (median); case: 3.87 (median)	NA	Yes
	7,660	8,695	154	12	NA	139	14	control: 3.07 (median); case: 3.88 (median)	NA	Yes
Cherskov et al. (2018) [21]	2,066	NA	NA	971	NA	NA	NA	NA	No ASD: 30.3 (9.1); ASD: 30.3 (9.1)	Yes
	26,263	NA	NA	160	NA	NA	NA	NA	No PCOS: 34.5 (8.5); PCOS: 35.5 (8.5)	Yes
	NA	8588	418	NA	12	NA	NA	NA	No PCOS: 27.9 (5.5); PCOS: 27.7 (5.2)	Yes
Bell et al. (2018) [16]	NA	458	NA	NA	NA	NA	NA	0– 3	PCOS: 31.2 (4.6); No PCOS: 30.3 (6.1)	Yes
Hisel-Gorrman et al. (2018) [27]	NA	556	NA	NA	NA	NA	NA	3–19	29.9 (IQR:28.3–31.7)	Yes
Cesta et al. (2020) [8]	NA	12,955	2,223	NA	NA	6,723	NA	0–40	20– 35 (83.1%)	Yes
Chen et al. (2020) [18]	NA	NA	323	NA	NA	881	NA	4–22	25–34 (PCOS: 63%; No PCOS: 65.5%)	Yes
Robinson et al. (2020) [9]	NA	194	NA	NA	NA	193	NA	NA	PCOS: 31.5 (4.7); All: 31.3 (5.9)	Yes
Total	43,971	44,125	41,442	1,227	13	8,073	38	0–40 years	Average range: 27–47 years	Yes

Read code was used as a diagnostic classification according to UK primary care practice standard. Women with PCOS report the number of PCOS females from studies reporting the association between PCOS and NPD in women themselves. Mothers with PCOS report the number of mothers with PCOS from studies reporting the association between maternal PCOS and NPD in children. Mothers with ASD report the number of mothers with ASD children from studies reporting the association between maternal PCOS and NPD in children; Women with ASD or ADHD report the number of women with ASD or ADHD from studies reporting the association between PCOS and NPD in women themselves. Children with ASD or ADHD report the number of children with ASD or ADHD from studies reporting the association between maternal PCOS and NPD in children.

*PCOS* polycystic ovary syndrome, *NPD* neuropsychiatric disorders, *HA* hyperandrogenemia, *ICD* international classification of diseases, *TMQ* Testosterone-related Medical Questionnaire, *NIH* national institute of health, *ASD* Autism spectrum disorder, *ADHD* attention deficit hyperactivity disorder, *CTD* chronic tic disorder, *PDD-NOS* pervasive developmental disorder––not otherwise specified, *SD* standard deviation, *IQR* interquartile range, *NA* not available.

### Quality assessment and publication bias

Using the AHRQ standards, most studies were classified as good quality (*n* = 11) or fair quality (*n* = 4) studies (Supplementary Table 1). Publication bias was assessed for primary outcomes in the studies reporting an association between maternal PCOS and ASD or ADHD in children. The symmetry pattern of funnel plots indicates an absence of publication bias (Supplementary Figure 1). Furthermore, no indication of small-study effects was observed for ASD or ADHD outcomes (Supplementary Table 2).

### Association between maternal PCOS and NPD in children

**Fig. 2:**
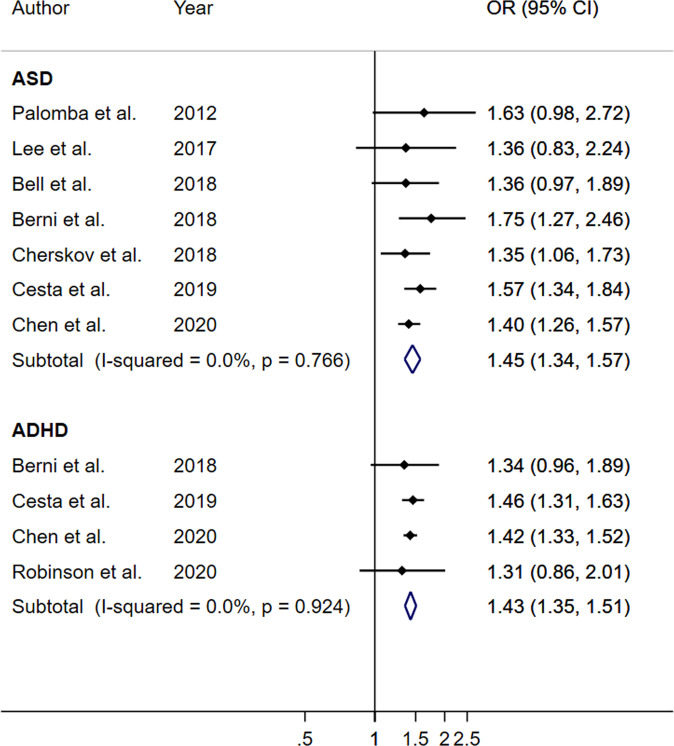
Maternal PCOS on the development of ASD and ADHD in children using the analysis of cohort studies. PCOS polycystic ovary syndrome, ASD Autism spectrum disorder, ADHD Attention deficit hyperactivity disorder.

In adjusted OR analysis, maternal PCOS was significantly associated with ASD (OR: 1.40; 95% CI: 1.297, 1.53; *p* < 0.001; *I*^*2*^ = 26.5%) and ADHD (OR = 1.42; 95% CI: 1.35, 1.49; *p* < 0.001; *I*^*2*^ = 0%) (Table 2). Furthermore, PCOS mothers had increased odds of developing ASD (OR = 1.45; 95% CI: 1.34, 1.57; *p* < 0.001; *I*^*2*^ = 0%) and ADHD (OR = 1.43; 95% CI: 1.35, 1.51; *p* < 0.001; *I*^*2*^ = 0%) without any significant presence of heterogeneity (Table 2, Fig. 2). In addition, mothers with PCOS had also increased odds of having CTD (OR = 1.44, 95% CI: 1.24, 1.68; *p* = 0.001; *I*^*2*^ = 0%), anxiety (OR = 1.33, 95% CI: 1.26, 1.41; *p* < 0.001; *I*^*2*^ = 0%), and other behavioral disorders (OR = 1.45, 95% CI: 1.37, 1.54; *p* < 0.001; *I*^*2*^ = 0%) in their children (Table 2). These associations were consistent between unadjusted and adjusted OR analysis (Table 2).

**Table 2:** Association between maternal PCOS and neuropsychiatric disorders in their children.

Neuropsychiatric disorders	*N*	Unadjusted association			Adjusted association^a^		
		*I*^*2*^	OR (95% CI)	*p* value	*I*^*2*^	OR (95% CI)	*p* value
ASD	12	28.4%	1.59 (1.47, 1.73)	<0.001	26.5%	1.40 (1.29, 1.53)	<0.001
ADHD	5	70.2%	1.54 (1.37, 1.73)	<0.001	0.0%	1.42 (1.35, 1.49)	<0.001
CTD	2	47.4%	1.56 (1.19, 2.03)	0.001	0.0%	1.44 (1.24, 1.68)	<0.001
Anxiety	2	0.0%	1.33 (1.26, 1.41)	<0.001	0.0%	1.33 (1.26, 1.41)	<0.001
Other behavioral symptoms	3	0.0%	1.45 (1.37, 1.54)	<0.001	0.0%	1.45 (1.37, 1.54)	<0.001
ASD in cohort studies	7	11.6%	1.57 (1.44, 1.72)	<0.001	0.0%	1.45 (1.34, 1.57)	<0.001
ADHD in cohort studies	4	33.9%	1.48 (1.35, 1.62)	<0.001	0.0%	1.43 (1.35, 1.51)	<0.001

*N* number of studies, *ASD* autism spectrum disorder, *ADHD* attention deficit hyperactivity disorder, *PCOS* Polycystic ovary syndrome, *CTD* chronic tic disorder, *OR* odds ratio, *CI* confidence interval.

^a^Adjusted association included adjusted ORs if available.

**Fig. 3:**
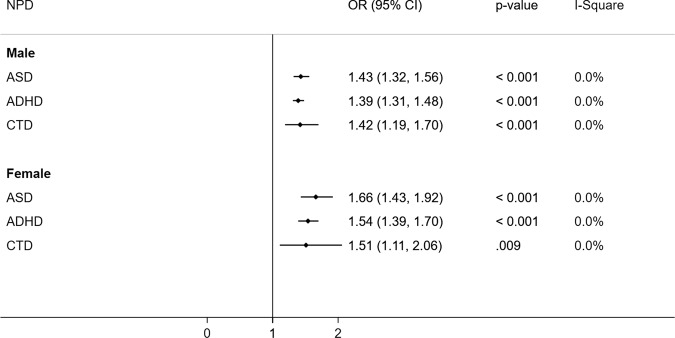
Association of maternal PCOS and NPD in children by their sex. PCOS polycystic ovary syndrome, NPD neuropsychiatry disorders, OR odds ratio, CI confidence interval.

In addition to anxiety, CDT, ASD, and ADHD, Chen et al. [18] also found that maternal PCOS with perinatal problems had an increased risk of sleeping disorders (hazard ratio, HR = 2.22, 95% CI: 1.69–2.92), eating disorders (HR = 1.90; 95% CI: 1.35–2.67), mood disorders (HR = 1.39; 95% CI:1.20–1.62), specific developmental disorders (HR = 2.26; 95% CI: 2.06–2.49), and intellectual disorders (HR = 2.91; 95% CI: 2.31–3.65) in the adjusted analysis and these associations remained significant even in PCOS mothers with no perinatal problems compared to mothers without PCOS. Although maternal PCOS was found to be significantly associated with NPD in both male and female children, the effect size was observed higher in females for ASD (1.66 vs. 1.43), ADHD (1.54 vs. 1.39), and CTD (1.51 vs. 1.42) than in males (Fig. 3).

### Subgroup and sensitivity analyses for ASD or ADHD in children

The magnitude of the association between maternal PCOS and ASD in children was highest in cohort design (OR = 1.45, *p* < 0.001, *I*^*2*^ = 0%) and UK (OR = 1.50, *p* < 0.001, *I*^*2*^ = 0%) studies while non-significant in the USA studies (OR = 1.15, *p* = 0.084, *I*^*2*^ = 0%). The effect sizes for the association between maternal PCOS and children ASD remained consistent in the analysis of restricted studies based on various study characteristics (Supplementary Table 3). Similarly, sensitivity analyses further yielded a consistent association between maternal PCOS and ADHD in children (OR ranges: 1.42–1.43) (Supplementary Table 3). Using meta-regression analysis, we did not find any maternal cofactors associated with pooled estimates of ASD or ADHD (Supplementary Table 4).

### Association between fetal testosterone exposure and autistic traits in children

Of five studies, three studies [11, 22, 23] used fetal amniotic measurements with radioimmunoassay, one study [25] used umbilical cord blood samples and another study [26] used maternal plasma levels with liquid chromatography-tandem mass spectrometry for measuring peri or prenatal testosterone exposure in children. Although prenatal testosterone exposure did not linearly correlate with autistic traits in children overall (*N* = 5, *r* = 0.15, *p* = 0.09), a weak correlation was observed (*N* = 3, *r* = 0.27, *p* = 0.037) after excluding studies that either used umbilical cord blood samples [25] or maternal plasma samples [26] or even excluding only Whitehouse at al study [25] (*N* = 4, *r* = 0.226, *p* = 0.04). Moreover, after excluding related studies such as Auyeung et al. studies [22, 23] (*N* = 3, *r* = 0.014, *p* = 0.70) and Baron-Cohen et al. study [11] (*N* = 2, *r* = 0.003, *p* = 0.95), no significant correlation was found without any heterogeneity (Table 3).

**Table 3:** Association between prenatal testosterone exposure and autistic traits in children.

	*N*	*I*^*2*^	Correlation	95% CI		*p* value
Overall	5	89.4%	0.148	−0.023	0.318	0.090
Excluding Xu et al. study	4	91.3%	0.162	−0.030	0.355	0.099
Excluding Whitehouse et al. study	4	90.4%	0.226	0.010	0.443	0.040
Excluding Whitehouse et al. and Xu et al. studies	3	93.0%	0.274	0.016	0.531	0.037
Excluding Whitehouse et al. and Barron et al. studies	3	75.9%	0.308	0.130	0.485	0.001
Excluding Xu et al., Barron et al., and Whitehouse et al. studies	2	0.0%	0.400	0.313	0.487	<0.001
Excluding Auyeung et al. studies	3	0.0%	0.014	−0.056	0.083	0.696
Excluding Auyeung et al. and Baron-Cohen et al. studies	2	0.0%	0.003	−0.089	0.095	0.947

*CI* confidence interval.

### Association between NPD and PCOS in women of reproductive age

Our pooled adjusted analysis based on three studies [13, 17, 21] demonstrated women with PCOS had higher odds of having ASD (OR = 1.88, *p* = 0.001). After including two additional studies [20, 36], the association between women with PCOS and ASD remained statistically significant (OR = 1.58, *p* = 0.001). In unadjusted OR analysis, PCOS was associated with ADHD (OR = 1.23, *p* < 0.001) and schizophrenia (OR = 1.67, *p* < 0.001) without a significant presence of heterogeneity even after adding data from an additional study [20]. However, the association between PCOS and ADHD was no longer significant in adjusted OR analysis (OR = 0.91, *p* = 0.069). In this study, PCOS women were also more likely to observe with anxiety (OR = 1.76 *p* < 0.001), and depression (OR = 1.92, *p* < 0.001). After compiling our meta-analysis findings with another meta-analysis study [37] on mental disorders, PCOS women consistently showed higher odds of having anxiety and depression, however with a significant presence of heterogeneity (Table 4).

**Table 4:** Association between PCOS and neuropsychiatric disorders in women of reproductive age.

Neuropsychiatric disorders	*N*	Unadjusted association				Adjusted association^a^		
		*I*^*2*^	OR (95% CI)	*p* value	*I*^*2*^		OR (95% CI)	*p* value
ASD	3	0.0%	2.31 (1.82, 2.93)	<0.001	15.4%		1.88 (1.38, 2.57)	<0.001
ASD^bc^	5	66.7%	1.93 (1.43, 2.61)	<0.001	51.7%		1.58 (1.22, 2.04)	0.001
ADHD^b^	2	0.00%	1.23 (1.13, 1.35)	<0.001	0.00%		0.91 (0.83, 1.01)	0.069
Schizophrenia	2	0.00%	1.67 (1.44, 1.94)	<0.001	0.00%		1.12 (0.94, 1.33)	0.199
Schizophrenia^b^	3	0.00%	1.72 (1.52, 1.94)	<0.001	17.7%		1.22 (1.04, 1.43)	0.014
Anxiety	2	97.10%	2.02 (1.56, 2.61)	<0.001	0.0%		1.76 (1.70, 1.83)	<0.001
Anxiety^b^	3	99.1%	1.86 (1.42, 2.43)	<0.001	97.5%		1.62 (1.35, 1.95)	<0.001
All anxiety^db^	8	96.4%	1.66 (1.38, 1.99)	<0.001	96.4%		1.66 (1.38, 1.99)	<0.001
Depression	2	99.10%	2.03 (1.45, 2.86)	<0.001	97.8%		1.92 (1.53, 2.42)	<0.001
Depression^b^	3	99.4	1.86 (1.37, 2.52)	<0.001	99.4		1.66 (1.18, 2.35)	0.004
All depression^db^	9	99.1%	1.86 (1.35, 2.56)	<0.001	99.1%		1.86 (1.35, 2.56)	<0.001

*ASD* autism spectrum disorder, *ADHD* attention deficit hyperactivity disorder, *PCOS* Polycystic ovary syndrome, *OR* odds ratio, *CI* confidence interval.

^a^Adjusted association included adjusted ORs if available.

^b^after including an additional study by Cesta et al. study;

^c^after including an additional study by Pohl et al.

^d^after including an additional meta-analysis study by Blay et al.

## Discussion

Our systematic review and meta-analysis based on a large number of studies showed a strong and similar magnitude of association between maternal PCOS and a range of NPD in their children. Moreover, our study also confirms that maternal PCOS is associated with the development of ASD and ADHD in their children. These findings suggest maternal PCOS is a risk factor for neurodevelopmental and behavioral disorders in children. To the best of our knowledge, our study for the first time showed a consistent association between maternal PCOS and NPD in children between males and females suggesting that children of PCOS mothers have higher odds of NPD regardless of their sex. However, fetal testosterone exposure in children was not found to be associated with autistic traits in our study. Altogether these findings indicate a combination of genetic and perinatal factors in PCOS mothers may contribute to NPD in their children regardless of sex––which in itself is interesting given the predominance of male children being diagnosed with ASD relative to females. Our analysis also confirms the higher odds of having NPD in women themselves suggesting that managing PCOS is essential for women’s health as well as for their children’s health.

In line with a previous meta-analysis [6], we confirmed that maternal PCOS is associated with children with ASD. The magnitude of the association between maternal PCOS and ASD in our study based on a relatively larger number of studies was lower than the effect size noted in the previous meta-analysis study [6]; however, our data closely corresponds to the effect size reported in the restricted analysis of good quality of studies. In support of our study, a recent review highlighted the role of environmental disruptive chemicals including androgens as having a role in the pathogenesis of ASD [38]. However, it is unclear whether treated or untreated PCOS mothers are more likely to be associated with ASD in children. Mamidala et al. showed that maternal hormonal interventions are associated with ASD [12] whereas Schieve et al. [15] did not find infertility treatments associated with ASD in children. However, a prior study reported that untreated PCOS mothers are more likely to be associated with ASD in children compared to treated PCOS mothers [16]. Hisle-Gorman found that ASD is strongly associated with maternal medication use rather than maternal PCOS diagnosis alone [27]. Our restricted analysis of cohort studies suggests maternal PCOS is associated with increased odds of developing ASD and ADHD. Furthermore, our sensitivity analyses produced highly consistent associations regardless of variations in ASD diagnosis, PCOS diagnosis, and study type. These findings suggest maternal PCOS is a risk factor for ASD and ADHD. However, our study yielded no association between maternal PCOS and ASD in the US studies than the studies from other geographical locations. This could be due to the inclusion of mostly self-reported PCOS diagnoses in the US studies [15, 16] compared to other regional studies. Self-reported gynecological conditions such as PCOS are often underreported due to recall bias and misclassification [9].

Our study further extends the previous knowledge that maternal PCOS does not only associate with ASD in children but also influences other neurodevelopment disorders such as ADHD, CDT, and other behavioral abnormalities. In our study, the strength of association between maternal PCOS and NPD in children was consistent throughout the spectrum of studies. In fact, the strength of association (crude OR: 1.45–1.59; adjusted OR: 1.40–1.45) obtained between maternal PCOS and NPD (including ASD, ADHD, CTD, other behavior symptoms) in our study was comparable to the effect size established for maternal age as a risk factor for ASD based on a large meta-analysis (crude relative risk = 1.52) [39]. Although these disorders shared comorbidity with ASD, previous studies [18, 19, 21] reported the associations of neurodevelopment disorders with maternal PCOS independent of ASD. We also observed a strong association between maternal PCOS and anxiety in children. Like our findings based on adjusted ORs, Chen et al. [18] confirmed that maternal PCOS even without obesity or perinatal problems was found to be associated with various NPD including ADHD, CTD, mood disorders, anxiety disorders, eating disorders, and other behavioral disorders. In addition, maternal PCOS with obesity or perinatal problems aggravate the odds of NPD in children. This suggests that the PCOS comorbidities that influence the increased risk of NPD in children should also be considered to stratify the risk of maternal PCOS on NPD for proper care. Although the etiology of NPD in children of mothers with PCOS is not fully understood, many factors may be considered for underlying mechanisms. Hormonal disturbances in PCOS impact neurobehavioral abnormalities by influencing the pathways that are involved in neurological processes and the formation of fetal brain development and regions associated with a wide range of NPD [9, 18, 19, 28]. Interactions of androgens with sex hormone genes, metabolic disorders, chronic inflammation, and altered gut-microbiota in PCOS mothers may affect fetal brain development explaining a variety of NPD in children [9, 18, 19, 28].

Neurodevelopmental disorders such as ASD and ADHD are often observed 4-10 times higher in males than females in the general population [40, 41]. In contrast, we observed slightly pronounced associations of maternal PCOS with NPD in females compared to males opposing extreme male brain theory of neurodevelopmental disorders in PCOS mothers. This is probably due to the high androgen exposure in utero in females. In addition, the genetic linkage between PCOS mothers with PCOS children may be another contributory factor for excessive risk of NPD in female children born to PCOS mothers [42]. Therefore, genetic polymorphism and epigenetic factors cannot be excluded for understanding mechanisms between maternal PCOS and NPD in their children.

Although prenatal exposure to the hyperandrogenic environment may also independently contribute to the development of a range of NPD in children [43], we did not find an association of fetal testosterone exposures with autistic traits using studies that measured fetal testosterone levels in amniotic fluid only. Contrary to our study, a number of studies measured fetal testosterone exposures in the umbilical cord or amniotic fluid and demonstrated a significant association with autistic associated behavioral features such as reduced eye contact, cognitive abilities, restricted interest, and social relationships, lower vocabulary size, and increased systemizing [44–50]. In addition, several studies used the 2D:4D ratio as an indirect measure of fetal testosterone exposure and reported the 2D:4D ratio associated with autistic traits [51, 52]. Kothari et al. also reported a lower 2D:4D ratio in daughters of mothers with lifetime eating disorders compared to daughters of women without eating disorders [24]. Altogether these findings indicate that the androgenic environment in early intrauterine life might be critical than perinatal testosterone exposure for causing autistic-like traits. Women with hirsutism or hyperandrogenism were also more likely to observe NPD in children [9, 14]. Consistent with previous studies [9, 37], our updated meta-analysis produced a strong association of PCOS with a broader spectrum of NPD in women themselves suggesting that PCOS has detrimental effects on various NPD in women themselves. In summary, our study confirms that maternal PCOS is associated with an increased odds of NPD in children independent of sex as well as in women themselves, and primarily due to maternal androgen exposure at early intrauterine life. However, the impact of timing of androgen exposure and their treatments on fetal life needs to be thoroughly examined in further studies.

There are limitations in our study. We could not perform sensitivity and subgroup analyses for all considered NPD other than ASD and ADHD due to a limited number of studies reporting all NPD. Although we analyzed data using both unadjusted and adjusted effect sizes due to variation in adjusted factors in different studies, our primary analyses of pooled adjusted estimates did not yield a significant presence of heterogeneity for any NPD supporting the robustness and generalizability of our study findings. The high number of screening studies indicates our efforts in comprehensive search by using relative terms used in published papers to not miss any potential studies on the topic. Typically, a meta-analysis may produce biased estimates possibly due to publication bias (negative studies or small-size studies are less likely to be published than positive or large studies). However, our publication bias exploration clearly indicated an absence of publication bias or small study effects. Moreover, most of the included studies are based on a large sample size indicating an absence of possible bias. Although studies from Europe fairly represent our study population, it is unlikely that the association confounded with geographic differences as most studies included in the meta-analysis were based on a confirmed standard diagnosis of PCOS and NPD. Furthermore, all associations had low or absence of heterogeneity as indicated by I^2^ (range 0%-26.5%) in the primary and sensitivity analyses. Moreover, our sensitivity and meta-regression analyses did not find any cofactors influencing the pooled effect sizes estimated in this study. Our methodological rigor and stratified analysis by sex yielded reliable and sex-specific findings to develop intervention strategies. To the best of our knowledge, our study is the first comprehensive meta-analysis involving ASD and other NPD in children associated with maternal PCOS based on a reasonably large number of good-quality studies with large sample sizes. For the first time, our study also produced pooled association estimates for NPD in children in relation to maternal PCOS separately for males and females. We also reported the longitudinal effect of maternal PCOS on the development of ASD and ADHD in children and produced correlation estimates between fetal testosterone exposure and autistic traits.

## Conclusion

Available evidence based on pooled analyses suggests maternal PCOS is a risk factor for NPD in children independent of their sex and psychiatric comorbidities. We recommend close monitoring of children of mothers with PCOS to provide early intervention. Based on updated evidence, our study also suggests early screening and management of women with PCOS or hirsutism for improving their own health related to NPD is critical. Further research is needed to understand the effects of PCOS treatments during pregnancy or infertility treatments on various NPD in their children. Understanding of potential mechanisms between maternal PCOS and NPD in children and identification of potential biomarkers is critically needed for developing prevention and intervention strategies.

## Supplementary information

Supplementary Figure 1

Supplementary Tables
