# Supplementary figures and images for: A systematic review and meta-analysis of the association between maternal polycystic ovary syndrome and neuropsychiatric disorders in children
Source: Transl Psychiatry. 2021 Nov 8;11:569. doi: 10.1038/s41398-021-01699-8 (PMC8575994; doi:10.1038/s41398-021-01699-8)

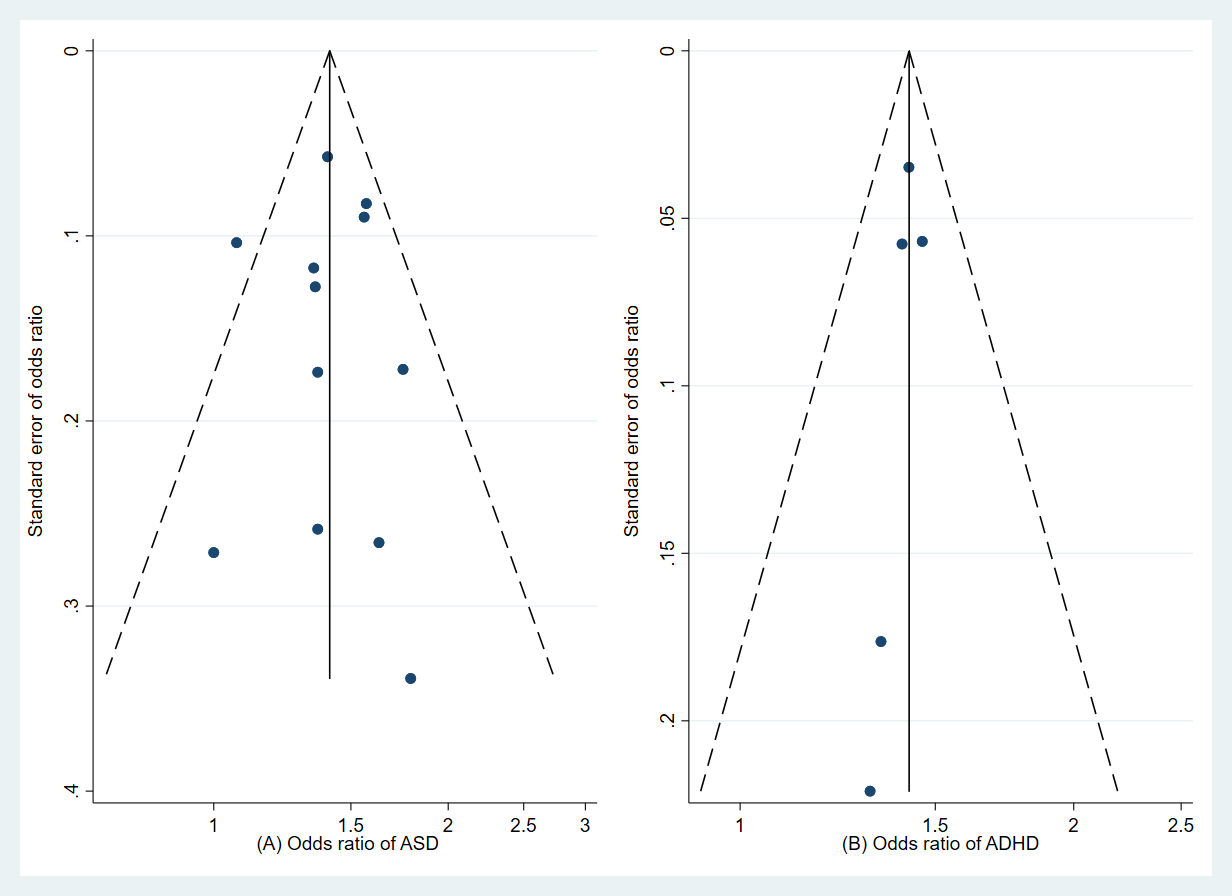

Supplement: Supplementary file 1 — Supplementary Figure 1 [file 41398_2021_1699_MOESM1_ESM.tif]
