# Supplementary material for: A systematic review and meta-analysis of the association between maternal polycystic ovary syndrome and neuropsychiatric disorders in children
Source: Transl Psychiatry. 2021 Nov 8;11:569. doi: 10.1038/s41398-021-01699-8 (PMC8575994; doi:10.1038/s41398-021-01699-8)
Supplement: Supplementary file 2 — Supplementary Tables [file 41398_2021_1699_MOESM2_ESM.docx]

Supplementary Table 1. Quality assessment of included studies in meta-analysis using Newcastle–Ottawa Scale

|  | **Selection** | | | | **Comparability** | **Exposure/Outcome** | | | **Total** | **AHRQ** |
| --- | --- | --- | --- | --- | --- | --- | --- | --- | --- | --- |
|  | q1 | q2 | q3 | q4 | q1 | q1 | q2 | q3 |  |  |
| **Case-control study** |  |  |  |  |  |  |  |  |  |  |
| Ingudomnukul  et al. (2007) [13] |  | * | * |  | * |  | * |  | 4 | poor |
| Palomba  et al.(2012) [14] | * | * | * | * | * | * | * | * | 8 | good |
| Mamidala  et al. (2013) [12] | * | * | * | * | * |  | * | * | 7 | good |
| Xu  et al. (2013) [26] | * | * | * | * | * | * |  |  | 6 | fair |
| Kosidou  et al. (2016) [19] |  | * | * | * | * | * | * | * | 7 | good |
| Kosidou  et al. (2017) [28] |  | * | * | * | * | * | * | * | 7 | good |
| Lee  et al. (2017) [35] |  | * | * | * | * |  |  | * | 5 | fair |
| Schieve  et al. (2017) [15] |  | * | * | * | * |  |  |  | 4 | poor |
| Cherskov  et al. (2018) [21] | * | * | * |  | * | * | * | * | 7 | good |
| **Cohort study** |  |  |  |  |  |  |  |  |  |  |
| Auyeung  et al. (2009) [22] | * |  | * |  | * |  | * | * | 5 | fair |
| Auyeung  et al. (2010) [23] | * |  | * |  | * |  | * | * | 5 | fair |
| Whitehouse  et al. (2012)* [25] | * |  | * | * |  | * | * | * | 6 | good |
| Baron-Cohen  et al. (2015) [11] |  | * | * |  | * | * | * |  | 5 | fair |
| Schieve  et al. (2017) [15] | * | * | * | * | * | * | * |  | 7 | good |
| Berni  et al. (2018) [17] | * | * | * |  | * | * |  | * | 6 | good |
| Hisel-Gorrman  et al. (2018) [27] | * | * | * |  | * | * | * | * | 7 | good |
| Cesta  et al. (2020) [8] | * | * | * |  | * | * |  | * | 6 | good |
| Chen  et al. (2020) [18] | * | * | * |  | * | * | * | * | 7 | good |
| Robinson et al. (2020) [9] | * | * | * |  | * | * | * | * | 7 | good |

**Abbreviations:** AHQR, agency for healthcare research and quality. *Quality of a cross-sectional study was also assessed using cohort study criteria of Newcastle–Ottawa Scale; q1-q4: questions.

Supplementary Table 2. Assessment of small-studies effects on ASD and ADHD in relationship with maternal PCOS

| **Outcomes** | **Statistical methods** | **N** | **Effect size** | **p-value** |
| --- | --- | --- | --- | --- |
| ASD | Egger’s RC  (95% CI) | 12 | -0.05  (-1.68, 1.57) | 0.94 |
|  | Kendall’s score* (SD) using Begg’s test | 12 | -2 (14.6) | 0.97 |
| ADHD | Egger’s RC  (95% CI) | 5 | -0.37 (-1.32, 0.58) | 0.31 |
|  | Kendall’s score* (SD) using Begg’s test | 5 | -8 (4.1) | 0.09 |

**Abbreviations:** RC, regression coefficient; CI, confidence interval; SD, standard deviation; PCOS, polycystic ovary syndrome; ASD, autism spectrum disorder; ADHD, attention deficit hyperactivity disorder; *continuity corrected p-values are reported.

Supplementary Table 3. Subgroup and sensitivity analyses for the association of maternal PCOS with ASD and ADHD in children

|  | **N** | **I^2^** | **OR** | **95% CI** | | **p-value** |
| --- | --- | --- | --- | --- | --- | --- |
| **ASD outcome** |  |  |  |  |  |  |
| **Study design** |  |  |  |  |  |  |
| Cohort | 7 | 0.0% | 1.45 | 1.34 | 1.57 | < 0.001 |
| Case-control | 5 | 59.3% | 1.31 | 1.07 | 1.59 | 0.007 |
| **Study type** |  |  |  |  |  |  |
| Population-based study | 9 | 42.8% | 1.39 | 1.26 | 1.54 | < 0.001 |
| Hospital-based study | 3 | 0.0% | 1.42 | 1.17 | 1.73 | < 0.001 |
| **Country** |  |  |  |  |  |  |
| USA | 4 | 0.0% | 1.15 | 0.98 | 1.34 | 0.084 |
| UK | 3 | 0.0% | 1.50 | 1.27 | 1.82 | < 0.001 |
| Others | 5 | 0.0% | 1.45 | 1.36 | 1.58 | < 0.001 |
| **Sensitivity analyses on restricted studies** |  |  |  |  |  |  |
| **After excluding few studies** |  |  |  |  |  |  |
| ASD^#^ | 11 | 32.4% | 1.41 | 1.29 | 1.55 | < 0.001 |
| ASD^$^ | 11 | 33.0% | 1.41 | 1.29 | 1.54 | < 0.001 |
| ASD^#$^ | 10 | 38.9% | 1.41 | 1.28 | 1.56 | < 0.001 |
| **PCOS diagnosis** |  |  |  |  |  |  |
| ASD without HA criteria for PCOS | 11 | 31.8% | 1.40 | 1.28 | 1.53 | <0.001 |
| ASD without self-reported criteria for PCOS | 9 | 38.4% | 1.43 | 1.29 | 1.58 | <0.001 |
| **Conversion of effect size** |  |  |  |  |  |  |
| Reported (ASD) | 7 | 0.0% | 1.32 | 1.16 | 1.51 | <0.001 |
| Computed (ASD) | 5 | 0.0% | 1.47 | 1.35 | 1.60 | <0.001 |
| **Good quality studies for ASD** | 9 | 37.3% | 1.41 | 1.29 | 1.54 | <0.001 |
| **ADHD outcome** |  |  |  |  |  |  |
| Cohort study | 4 | 0.0% | 1.43 | 1.35 | 1.51 | <0.001 |
| Excluding studies with self-reported PCOS | 4 | 0.0% | 1.42 | 1.35 | 1.49 | <0.001 |
| Converted (ADHD) | 3 | 0.0% | 1.43 | 1.35 | 1.51 | <0.001 |

**Abbreviations:** ASD, autism spectrum disorder; ADHD, attention deficit hyperactivity disorder; PCOS, Polycystic ovary syndrome; HA, hyperandrogenemia; OR, odds ratio; CI, confidence interval; NSD, not sufficient data.

**Note:** ^#^ after excluding Mamidala et al. study which reported PCOS cases with hormonal treatment; ^$^, after excluding Bell et al. study using autism spectrum quotient tool for ASD diagnosis; #^$^, After excluding both Mamidala et al. and Bell et al. studies.

Supplementary Table 4. Associations of maternal and child characteristics with ASD and ADHD in children

| **Maternal-fetal characteristics** | **ASD** | | | | **ADHD** | | |
| --- | --- | --- | --- | --- | --- | --- | --- |
|  | **N** | **Odds ratio**  **(95%CI)** | **p-**  **value** | **N** | | **Odds ratio**  **(95%CI)** | **p-**  **value** |
| Follow up years | 7 | 1.00 (0.999, 1.02) | 0.73 | 5 | | 1.00 (0.99, 1.01) | 0.59 |
| Gestational age  (in cases) | 3 | 0.99 (0.81, 1.20) | 0.57 | NSD | | NSD | NSD |
| Maternal age | 7 | 1.01 (0.97, 1.06) | 0.54 | 3 | | 0.99 (0.46, 2.12) | 0.86 |
| Children age | 4 | 1.01 (0.90, 1.12) | 0.77 | NSD | | NSD | NSD |
| Gender: females | 9 | 1.01 (1.00, 1.01) | 0.14 | 4 | | 1.00 (0.98, 1.02) | 0.80 |
| BMI | 3 | 0.88 (0.07, 11.68) | 0.64 | NSD | | NSD | NSD |
| HTN | 3 | 0.99 (0.67, 1.49) | 0.99 | NSD | | NSD | NSD |
| DM | 3 | 0.96 (0.82, 1.13) | 0.22 | NSD | | NSD | NSD |
| Gestational DM | 3 | 0.98 (0.69, 1.39) | 0.60 | NSD | | NSD | NSD |

**Abbreviations:** ASD, autism spectrum disorder; ADHD, attention deficit hyperactivity disorder; CI, confidence interval; BMI, body mass index; HTN, hypertension; DM, diabetes mellitus; NSD, not sufficient data.
